# Six-helix bundle completion in the distal C-terminal heptad repeat region of gp41 is required for efficient human immunodeficiency virus type 1 infection

**DOI:** 10.1186/s12977-018-0410-9

**Published:** 2018-04-02

**Authors:** Dehua Liu, Hongyun Wang, Mizuki Yamamoto, Jiping Song, Rui Zhang, Qingling Du, Yasushi Kawaguchi, Jun-ichiro Inoue, Zene Matsuda

**Affiliations:** 1 Laboratory of Structural Virology and Immunology, Institute of Biophysics, Chinese Academy of Sciences, Beijing, China; 2 Research Center for Asian Infectious Diseases, Institute of Medical Science, The University of Tokyo, 4-6-1 Shirokanedai, Minato-ku, Tokyo, 108-8639 Japan; 3 Division of Molecular Virology, Department of Microbiology and Immunology, Institute of Medical Science, The University of Tokyo, Tokyo, Japan; 4 Division of Cellular and Molecular Biology, Institute of Medical Science, The University of Tokyo, Tokyo, Japan; 5 Present Address: The Chinese University of Hong Kong, Sha Tin, Hong Kong China

**Keywords:** Human immunodeficiency virus type 1, Envelope protein, Membrane fusion, Fusion pore, Heptad repeat, Six-helix bundle, Split luciferase, Split green fluorescent protein

## Abstract

**Background:**

The native pre-fusion structure of gp120/gp41 complex of human immunodeficiency virus type 1 was recently revealed. In the model, the helices of gp41 **(**α6, α7, α8, and α9) form a four-helix collar underneath trimeric gp120. Gp41 is a class I fusion protein and mediates membrane fusion by forming a post-fusion structure called the six-helix bundle (6HB). The comparison of the pre- and post-fusion structures revealed the large conformational changes in gp41 during the antiparallel packing of the N- and C-terminal heptad repeats (NHRs and CHRs) in membrane fusion. Several mutagenesis studies of gp41 performed in the past were interpreted based on 6HB, the only available structure at that time. To obtain an insight about the current pre-fusion structural model and conformational changes during membrane fusion, alanine insertion mutagenesis of the NHR, CHR and connecting loop regions of HXB2 gp41 was performed. The effects of mutations on biosynthesis and membrane fusion were analyzed by immunoblotting and fusion assays, respectively. The extent of membrane fusion was evaluated by split luciferase-based pore formation and syncytia formation assays, respectively.

**Results:**

Consistent with the current structural model, drastic negative effects of mutations on biosynthesis and membrane fusion were observed for NHR, loop, and proximal regions of CHR (up to amino acid position 643). The insertions in α9 after it leaves the four-helix collar were tolerable for biosynthesis. These CHR mutants showed varying effects on membrane fusion. Insertion at position 644 or 645 resulted in poor pore and syncytia formation. Efficient pore and syncytia formation almost similar to that of the wild type was observed for insertion at position 647, 648 or 649. However, recovery of virus infectivity was only observed for the insertions beyond position 648.

**Conclusions:**

The mutagenesis data for HXB2 gp41 is in agreement with the recent pre-fusion structure model. The virus infection data suggested that fusion pores sufficiently large enough for the release of the virus genome complex are formed after the completion of 6HB beyond position 648.

**Electronic supplementary material:**

The online version of this article (10.1186/s12977-018-0410-9) contains supplementary material, which is available to authorized users.

## Background

**Fig. 1:**
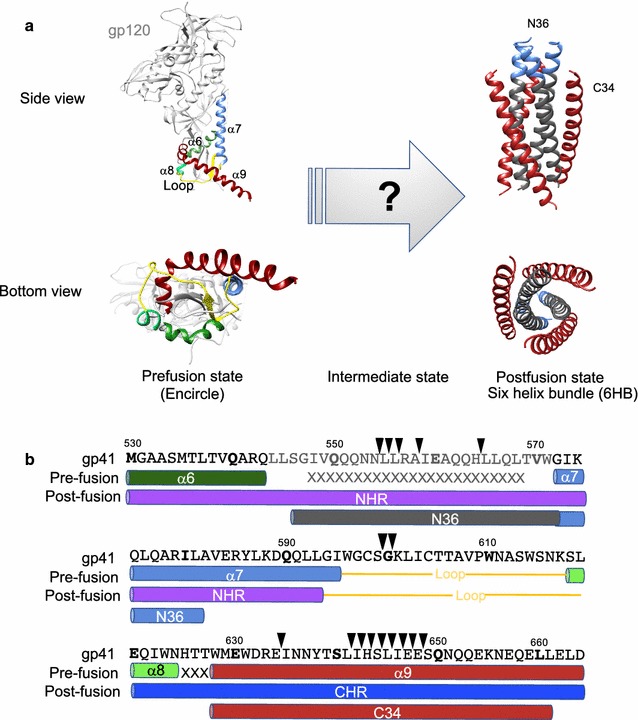
Conformational changes in Env during membrane fusion. **a** Pre-fusion and post-fusion structures of gp41. Left panel: Schematic representation of the prefusion structure of Env based on the structure of BG505 SOSIP.664, as determined by CryoEM (PDB 4TVP). The upper and lower images depict the side and bottom (from the viral membrane) views, respectively. The main chain of the gp120 subunit is shown in gray, and the gp41 subunit is shown in various colors corresponding to different α-helices. The loop between the NHR and CHR is shown in yellow. Note that only a monomer of Env is shown in the left panel, and bound antibodies PGT122 and 35O22 are removed for clarity. The N- and C- terminus of gp120 are wrapped in the center of the complex formed by gp41 [7]. Right panel: the post-fusion structures of the six-helix bundle (6HB) of gp41 (PDB 1AIK). NHR and CHR are shown in the same color assignment as in the left panel. The side and bottom views are shown. The images were generated by the University of California, San Francisco Chimera program. **b** The alanine insertion mutants used in this study. The insertion sites for alanine are indicated by arrowheads above the sequence of gp41. The sequence and secondary structures of HIV-1 gp41 are indicated. Both pre-fusion and post-fusion secondary structures are colored corresponding to **a**. Cylinders represent α-helices, and the disordered regions are indicated by “x”. The regions corresponding to N36 and C34 are also shown. Mutants are named by the position of the inserted alanine residue (such as 645+A). The numbering is based on HXB2 Env

The envelope protein of human immunodeficiency virus type 1 (HIV-1) is composed of gp120 and gp41. The gp120 subunit recognizes the receptor and co-receptor expressed on target cells and determines the host range of HIV-1 [1–3]. The gp41 subunit is a transmembrane protein and mediates membrane fusion between the viral and cellular membranes, an essential initial step of HIV-1 infection [4–6]. Recent structural analyses of BG505 SOSIP HIV-1 Env revealed the native pre-fusion state of gp120/gp41 complex [7, 8]. In this structure, the four helices of gp41 **(**α6, α7, α8 and α9) form a collar underneath trimeric gp120. Trimeric gp41 non-covalently associates with a gp120 trimer by encircling the N- and C-terminus of gp120 (Fig. 1a). Thus, gp120 and gp41 have a large interface between them and this intimate structural arrangement is believed to be responsible for the transmission of conformational changes of gp120 and gp41 during membrane fusion. The comparison of the pre-fusion structure with that of post-fusion gp41, called the six-helix bundle (6HB), revealed the presence of dynamic conformational rearrangements during membrane fusion such as loop to helix conversions, similar to those of influenza HA2 [7–12]. However, the details of the conformational changes remain elusive because the intermediate forms of gp41 remain unknown.

The gp41 subunit is a member of the class I fusion proteins, and the progress of membrane fusion is tightly linked with the formation of 6HB. After the interaction of gp120 and the receptor/co-receptor complex, changes of the gp120–gp41 interaction are expected to lead to exposure of the N-terminal heptad repeat (NHR) region of gp41. The C-terminal heptad repeat (CHR) bends over onto the grooves formed by the exposed trimeric NHRs to form 6HB [10, 11, 13, 14]. This packing brings the viral and cellular membranes closer, leading to the initial fusion pore formation via a hemifusion intermediate [15–19]. The current data suggested that the initial fusion pore is generated before completion of 6HB formation [4, 13, 20]. The stabilization and enlargement of the initial labile fusion pore to achieve full fusion require energy released by the completion of 6HB [4, 20–22].

In the past, extensive mutagenesis of gp41 such as alanine scanning mutagenesis [23–26] has been performed to reveal the structure–function relationship of gp41. The results were mainly interpreted based on the then-available structure of gp41, i.e., the post-fusion state structure (6HB) [10, 11], as the pre-fusion structure of gp41 was unknown. Because the structure model of gp41 in the native (pre-fusion) state complexed with gp120 became available recently (Fig. 1a), we revisited the issue of the structure–function relationship of HXB2 gp41. Currently, structural models are available for R5 tropic Env, and the structure of X4 tropic HXB2 Env has not been determined experimentally. However, the relatively high sequence homology of different gp41 variants suggests that the structure of HXB2 gp41 is similar to that of the currently available models.

We employed alanine insertion mutagenesis rather than alanine substitution. Insertional mutagenesis is expected to introduce more drastic structural alterations than point mutations, but alanine insertion can be accommodated to some extent dependent on the protein structure [27]. Thus, an insertional approach may probe the structural flexibility more efficiently than the single alanine substitution approach. Furthermore, if an insertion is tolerated and does not disturb the biosynthesis of HIV-1 Env extensively, the mutant may provide an insight into the critical boundary of 6HB formation required for membrane fusion because an insertion will result in the deregistration of α-helices in 6HB beyond the insertion point. We chose an alanine residue for insertion because it can be well-tolerated in an α-helix, which is a hallmark structure of class I fusion proteins such as gp41. We subjected the NHR, CHR, and connecting loop regions of gp41 to our mutagenesis protocol.

To obtain information on the progression of membrane fusion, particularly during the early phase, we employed our split luciferase-based fusion assay, called the dual split protein (DSP) assay [28–32]. The DSP assay can quantitatively evaluate the extent of fusion pore formation and content mixing by measuring signals from re-associated split *Renilla* luciferase (RL) pre-expressed in the effector and target cells prior to membrane fusion. Application of the DSP assay to the analysis of membrane fusion of herpes simplex virus successfully revealed the difference in the early phase of membrane fusion in the mutants [33]. We also used a classic syncytia formation assay that could provide information on the whole process of membrane fusion, including fusion pore formation, enlargement of fusion pores, and the merging of the involved cells. By comparing the results of these two assays, we could evaluate the effects of a mutation on different stages of membrane fusion, i.e., from initial formation to subsequent growth of fusion pores in a relatively simple experimental setting. We also evaluated the effect of mutation on virus infectivity by one round of pseudo-typed HIV-1 infection.

Consistent with the current pre-fusion gp120/gp41 structure model, most insertions in NHR and the loop region negatively affected biosynthesis of Env. For the relationship of fusion pore growth and the generation of 6HB, our results are consistent with previous results: (i) the fusion pore is formed before the completion of 6HB formation [13], and (ii) the progress of 6HB formation toward its C-terminus is necessary for the enlargement of the fusion pore [4]. Our data for virus infection suggest that the zipping of CHR (α9) in 6HB beyond position 648 is necessary to generate a pore sufficiently large to allow the release of the virus genome complex.

## Results

### Insertion of an alanine residue in the NHR and loop portions more negatively affected the biosynthesis of Env than insertions in CHR

To probe the structure–function relationship of gp41, we generated alanine insertion mutants of gp41 by introducing one alanine residue at a time in the coding sequences of NHR, CHR, and the connecting loop between them (Fig. 1b). The mutants were named by the position of the inserted alanine residue. For example, the mutant 645+A had one alanine inserted between residues 644 and 645 (numbering is based on HXB2 Env). Given the more intimate interaction of the NHR and loop region of gp41 with the inner domain of gp120 in the current pre-fusion structural model, we expected to generate more biosynthesis-defective mutants in these regions of gp41, as the alanine insertion is more detrimental than the alanine substitution. Therefore, we made fewer mutations in the NHR and loop regions. For the CHR region, we focused on the region of α9 near its exit from the four-helix collar (residue 642–649). We generated five NHR mutants, two loop mutants, and nine CHR mutants (Fig. 1b).

**Fig. 2:**
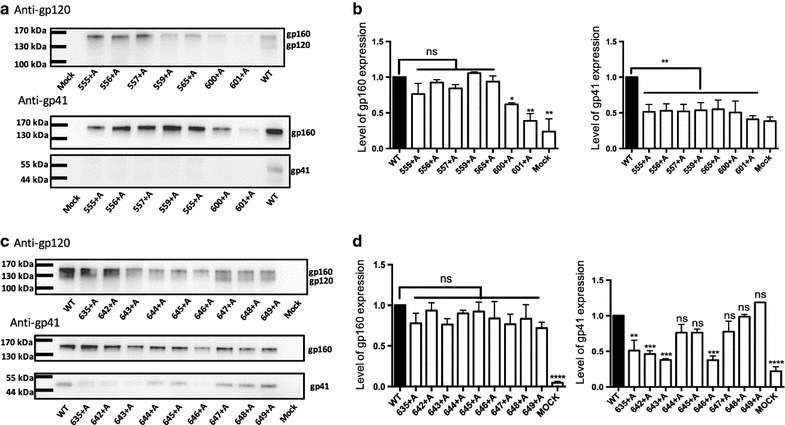
The protein profiles of Env mutants. The expression and processing of Env were analyzed by immunoblotting. Representative results of immunoblotting analysis of Env with NHR and loop mutants (**a**) and CHR mutants (**c**) expressed in the transfected 293FT cells probed with goat anti-gp120 or with Chessie 8 anti-gp41 antibodies are shown. The gp160 and gp41 bands were then quantified (**b** and **d**) by measuring the intensity of bands on the anti-gp41 immunoblot. The expression levels were normalized to that of the wild-type protein. Statistical analysis was performed using one way ANOVA. ****p < 0.0001, ***p < 0.001, **p < 0.01, *p < 0.05; ns indicates no statistically significant difference when compared with the WT protein

Because the proper processing of gp160 into gp120 and gp41 is a marker of proper biosynthesis of HIV-1 Env and a prerequisite for fusion activity, we analyzed the protein profiles of the mutants by immunoblotting using anti-gp120 and anti-gp41 antibodies (Fig. 2). For the loop mutants (600+A and 601+A), the expression level of gp160 was reduced. Furthermore, these mutants also showed decreased levels of gp41, which indicates defective processing of gp160 into gp120 and gp41. For insertion mutants in the NHR region (555+A, 556+A, 557+A, 559+A and 565+A), the expression level of gp160 was relatively retained, whereas the processing of gp160 was severely impaired (Fig. 2a and b).

For insertion mutants in the CHR region, the expression level of gp160 was similar to that of the wild-type protein for most of the mutants. However, the processing of the 635+A, 642+A, 643+A, and 646+A mutants that locate in the four-helix collar was significantly reduced. Other insertions closer to the exit of α9 from the four-helix collar, such as 644+A, 645+A, 647+A, 648+A and 649+A, showed comparable gp160 processing to that of the wild-type protein. Taken together, our results showed that the insertion in the NHR, loop, and proximal half of the CHR regions (up to 643) that have a closer interaction with gp120 affected the biosynthesis of HIV-1 Env more severely than the insertion in the distal portion of the CHR region. These aspects seem to be well accounted for by the current pre-fusion structural model.

### Mutants with insertion of an alanine residue in the distal portion of CHR of gp41 retained fusion activity

**Fig. 3:**
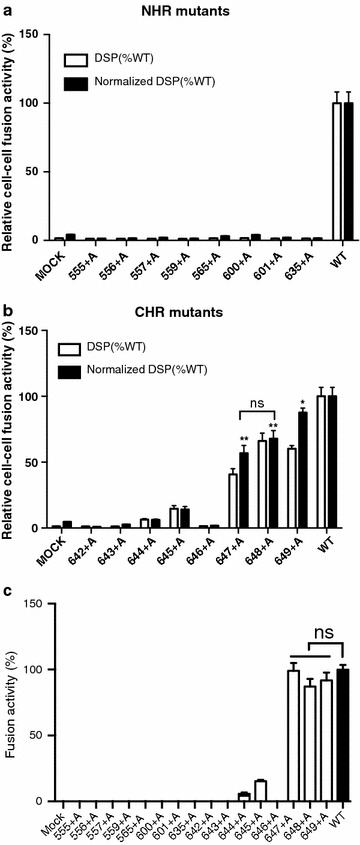
Fusion assay of alanine insertion mutants of gp41. **a** and **b** The DSP assay was performed at 2 h after coculture to examine the fusion activities of mutants. Surface expression levels of mutant Envs were determined by CELISA, and the fusion activity measured by the DSP assay (open bar) was normalized to the surface expression level (dark bar). Error bars represent the standard deviation of the results of triplicate experiments. The results for NHR and loop mutants are shown in **a**. **b** The results for CHR mutants. Statistical analysis was performed using *t* test. **p < 0.01 and *p < 0.05 when comparison of the indicated mutant with wild type was done. ns indicates no significant differences (*p* > 0.05) when 647+A and 648+A were compared. **c** Syncytia formation assays were used to evaluate the fusion activities of the mutants. The number of nuclei in the syncytia was divided by the total number of the nuclei in the field. Five randomly chosen fields were used to measure the value. The degree of syncytia formation is shown as a percentage (the wild-type was set as 100%). Statistical analysis was performed using *t* test. ns indicates no significant differences (*p* > 0.05) when compared with the value of the wild type

Next, each mutant was evaluated for its function in cell–cell fusion assays to evaluate the impact of the alanine insertion. First, a fusion assay using co-cultures of the effector and target cells expressing the split luciferase-based reporter protein, i.e., the DSP, was employed to evaluate the rate of fusion pore formation and context mixing between the effector and target cells by measuring the activities of re-associated split RL [28–30, 34]. All the DSP activities were normalized to the surface expression level of Env determined by CELISA. As expected and consistent with the results of the immunoblotting analysis (Fig. 2a and b), most of the insertions in the loop and NHR regions resulted in severely impaired recovery of RL activity, indicating severe impairment of fusion pore formation in these mutants (Fig. 3a).

For mutations in the CHR region, insertion in the proximal portion of CHR (635+A, 642+A and 643+A) and one mutation in the distal portion of CHR (646+A) resulted in severely impaired DSP activity (Fig. 3b). Again, this phenotype was consistent with the protein profile of poor processing of gp160 into gp120 and gp41 (Fig. 2c and d). Mutants 644+A and 645+A showed decreased activity of RL in the DSP assay, indicating poor fusion pore formation. The 647+A, 648+A and 649+A mutants showed relatively high RL activity, but they were still lower than that of the wild-type protein (Fig. 3b). There was no difference in the normalized DSP activity between 647+A and 648+A.

Because the presence of mutants with no defects in fusion pore formation, but defective enlargement of the fusion pores, has been demonstrated [35], we next performed syncytia formation assays to further investigate the entire process of membrane fusion in each mutant. Transient transfection of the expression vector for each HIV-1 Env mutant in 293CD4 cells was performed as previously described [34]. The degree of syncytia formation was measured by dividing the number of nuclei contained in syncytia by the total number of nuclei in the examined fields [36, 37]. The results are shown in Fig. 3c. Overall, there was a good correlation between the results of the DSP activity assay and degree of syncytia formation. For example, consistent with the null DSP activity, cells transfected with NHR and loop region mutants showed no syncytia formation (Fig. 3c). For the CHR mutants, insertions in the proximal portion of CHR tended to have more negative effects on syncytia formation activity than insertion in the distal portion of CHR. The 644+A and 645+A mutants with lower DSP activity showed a decreased level of syncytia formation. The mutants that showed comparable DSP activity to that of the wild-type protein (647+A, 648+A and 649+A) had a degree of syncytia formation similar to that of the wild-type protein (Fig. 3c).

**Fig. 4:**
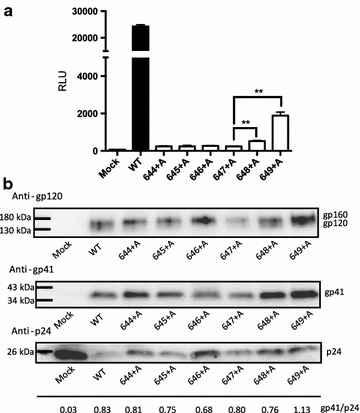
Pseudotype virus infection assay. **a** Infectivity of pseudotype virus bearing mutant Env was evaluated by measuring the reporter (n-luc) activity after 48 h of infection. The CHR mutants, 644+A, 645+A, 646+A, 647+A, 648+A, 649+A, were tested together with the wild type. Error bars represent the standard deviation of the results of triplicate experiments. Statistical analysis was performed using t test. **p < 0.01 when compared with 647+A. **b** The protein profiles of VLPs of CHR mutants were analyzed by immunoblotting. Anti-gp120, gp41 and p24 antibodies were used to detect the respective bands. The Env expression vector was omitted for mock transfection. The ratio of the band intensities of gp41 and p24 was indicated under the immunoblot

To examine the effect of the mutations on virus infection, we performed a one-round pseudotyped virus infection assay using some CHR mutant Envs. The representative results are shown in Fig. 4a. Only 648+A and 649+A Env showed a low level of infectivity, but the other tested mutants failed to achieve efficient infection. Notably, mutant 647+A that showed pore formation and syncytia formation similar to that of the wild type failed to infect the target cells. This failure of infection by 644+A, 645+A, 646+A and 647+A was not due to poor incorporation of Env into virions, because a similar amount of gp41 as the wild type was observed for these mutants (see the gp41/p24 ratio in Fig. 4b).

## Discussion

**Fig. 5:**
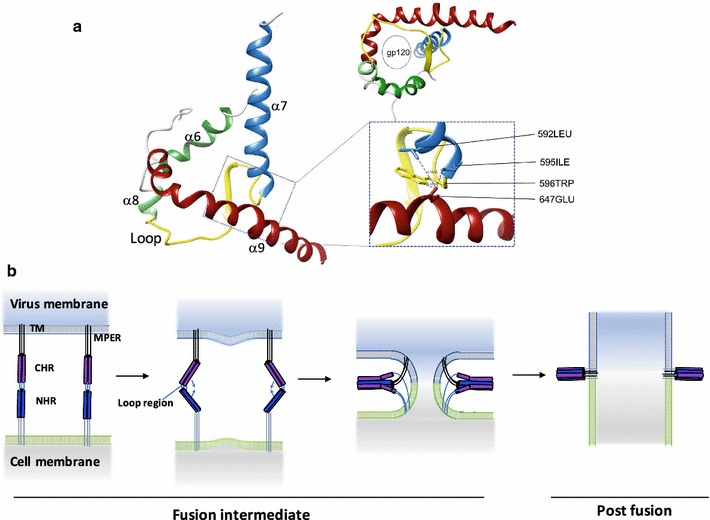
Schematic representation of the virus and cell fusion. **a** Prefusion structure of gp41. Side (left panel) and bottom (right upper panel) views of gp41 are shown. The four-helix collar (α6–α9) of gp41 encircled gp120 (gp120 is indicated as a dotted circle). Close-up view of gp41 around residue 647 is shown in the right lower panel. The distances between residues 647 and residues 592 (4.786 Å), 595 (3.199 Å), and 596 (4.829 Å) are indicated. **b** Schematic description of conformational changes in gp41 during membrane fusion based on our data. After the fusion peptide (FP) reached the host-cell membrane, packing of CHR into the NHR grooves was initiated near the connecting loop region between the CHR and NHR. Pairing of residues in the CHR located before residue 647 may be sufficient for generation of initial unstable fusion pores. Residues beyond 647 of the CHR needed to interact with the NHR to achieve pore enlargement. The cytoplasmic (CT) domain, transmembrane (TM) domains, fusion peptide (FP), membrane proximal external region (MPER), NHR, CHR, and loop region are indicated

We performed alanine insertion mutagenesis to probe the structure–function relationship of HXB2 gp41. Our assumption is that structure of HXB2 Env may be similar, if not identical, to the structure of BG505SOSIP. Similar to the previous studies [4, 25], mutations in the NHR and loop regions of gp41 tend to be defective in the biosynthesis of Env. An alanine residue insertion not only changed the amino acid residue at the insertion point to an alanine residue, but also induced a shift in the primary sequence and could introduce a rotational shift of 100° around the axis in the α-helix of the protein. This type of structural alterations seems to not be tolerable due to the recent structural model of the native gp120/gp41 complex [7–9], in which a trimeric coiled coil of NHRs forms a central hydrophobic core structure that interacts with the inner domain of gp120. The loop region of gp41 also has intimate hydrophobic interactions with gp120 by forming the base of a four-helix collar of gp41 (Fig. 5a). Alanine insertion is also not tolerable in the loop region.

The results showed that alanine insertion mutations in the distal portion of CHR were less damaging to biosynthesis. In the current structural model of the pre-fusion status, α9 leaves the bottom of the four-helix collar (formed by α6, α7, α8, and α9 of gp41) that wraps around the N- and C-terminus ends of the gp120 at position 647; therefore, the α9 residues beyond position 648 have less interaction with trimeric gp120 (Fig. 5a). This structural arrangement may account for the tolerance of mutations in the distal region of CHR (α9) for biosynthesis. Similar tolerance against mutations was observed in previous studies [4, 26].

It has been shown that the initial pore is formed through partial formation of 6HB [20]; these pores are labile and cannot progress to the stage of enlargement until the zipping proceeds towards the distal ends [4, 16, 19, 20]. The results of cell–cell fusion assays performed by several groups with alanine substitutions are available. For example, mutation studies of the **a** and **d** positions of HR2 by Markosyan et al. suggested that the pore enlargement gradually progresses during the formation of 6HB toward C-terminus end of CHR [4]. They observed slight gain of fusion activity for mutant 645, and larger recovery was observed for mutant 649. Diaz-Aguilar et al. observed good recovery of fusion after substitution of position 647, though with some fluctuation in fusion activities in position 643 [26]. We detected slight recovery of DSP activities for 644+A and good recovery for 647+A. Although the reporters used in these studies were different (dye, viral proteins, or split protein), the obtained results are similar. Our results for the CHR mutants showed that the fusion pore can be formed by partial zipping of 6HB until residue 644 and more efficient pore formation occurred when zipping progressed towards the C-terminus direction of CHR.

Different from the above cell–cell fusion assays, a previous virus infectivity assay of the alanine substitution mutants did not reveal such a clear boundary of change in infectivity [26]. The analysis of insertion mutants in our virus infection assay, however, showed that the critical boundary for infectivity was located between position 647 and 648; we found that the 647+A mutant failed to establish infection, whereas the 648+A mutant virus was able to infect target cells. The difference in the two studies may arise from the differences in the types of mutations. An alanine substitution may be more tolerable by only altering the local structure of 6HB and may not prevent the zipping beyond the mutation site.

Our alanine insertion, however, seems to have a more drastic impact on the zipping of 6HB because the CD analysis using the 647+A version of C34 with N36 failed to show the typical α-helical CD profile (Additional file 1: Fig. S1). Interestingly, the infectivity of the virus did not correlate well with the pore formation activities estimated by our DSP assay. This was probably because the size of our split reporter proteins was much smaller than the virus genome complex and could pass through the pores. Therefore, the fusion pores in 647+A mutant were of sufficient size to allow reporter proteins, but not the virus genome complex, to pass. However, since our assay could not directly evaluate the size of the formed fusion pores, alternative possibilities such as short half-life of the fusion pores made by mutant Env or the impact of the differences in the stability of mutant Env cannot be excluded.

In addition to the difference in the required pore size for the reporter proteins and viral genome, the limited number of available Env molecules on virions compared with the cell surface may be the reason for the more severe defect in the virus infection assay. Different from a previous study [26], we did not observe any recovery or compensation of defective cell–cell fusion activities in our virus assay. This could be due to the greater deterioration with our insertion mutation compared with a point mutation as discussed above. It will be also interesting to determine whether our findings in X4 tropic HXB2 can be replicated in other X4 strains or in R5 tropic Env, as one previous study indicated potential differences between the HXB2 and JRFL strains [26].

Based on our data, a schematic diagram of the progress of membrane fusion during virus infection is shown in Fig. 5b. When zipping proceeds beyond position 647 of CHR, the energy released becomes sufficient to produce larger fusion pores for the release of the virus genome. After further zipping of 6HB proceeds, membrane fusion proceeds, eventually leading to full fusion. This position of critical zipping coincidentally mapped to position 647 where a9 leaves the four-helix collar of gp41.

The exact nature or structure of the intermediate gp41 and its relationship with the lipid bilayer are still unclear; however, 644+A or 645+A mutant may be a useful model to investigate the elusive intermediate status of gp41. In addition to the structure of 6HB, when the pre-fusion structure of the gp120/gp41 is considered, the region around residue 647 may be important for regulation of the initial conformational change of gp41. For example, the 647 E residue may be involved in the interaction with residues within α7, such as 592, 595, and 596 (Fig. 5a). The insertion at position 647 may affect these intra-gp41 interactions and the initial conformational changes in gp41 [19]. Further mutations in α7 and α9 in future may shed light on this aspect.

Our analysis using the DSP reporter is relatively simple and does not require a very sophisticated laboratory setup. The analysis of the early stage of membrane fusion will provide useful information to further characterize the detailed mechanisms of membrane fusion mediated by HIV-1 Env. These analyses may lead to the design of rational fusion inhibitors that act on HIV-1 infection at its earliest stage.

## Conclusions

Analyses of the effects of alanine-insertion mutants of HXB2 gp41 for their biosynthesis and fusion activities in both cell–cell and virus-cell fusion assays were performed. The results suggested that HXB2 gp41 may have a structural arrangement similar to the currently available structural model based on R5 tropic BG505 Env. Our data implies that the fusion pore can be generated by partial 6HB completion. However, completion of 6HB beyond position 647 may be a prerequisite for fusion pore enlargement sufficient for the virus to acquire infectivity.

## Methods

### Plasmid construction

The HXB2 envelope gene sequence used in this study was codon-optimized for mammalian cell expression [34]. A QuickChange site-directed mutagenesis kit (Stratagene, La Jolla, CA, USA) was used to generate the mutants in this study. Briefly, the plasmid pHIV-env-opt HXB2, which contains the 2.6-kb *Sal*I-*EcoR*I fragment corresponding to HXB2 Env, was used as a template. Complementary oligonucleotide pairs containing an insertion codon for the alanine residue, GCA, were used. The mutant identified by sequencing was cloned back into the pHIV-env-opt HXB2 as the *BsrG*I and *BamH*I fragment. After cloning, the entire *Sal*I-*EcoR*I region including the vector junction was verified by sequencing. Polymerase chain reaction (PCR) was performed using Pfu Turbo (Stratagene).

### Cell culture and transfection

293FT cells (Invitrogen, Carlsbad, CA, USA) or 293CD4 cells (293 cells constitutively expressing human CD4) [37] were grown in Dulbecco’s modified Eagle’s medium (DMEM) supplemented with 10% fetal bovine serum (FBS). Cells were cultured in a 5% CO_2_ in a humidified incubator (Sanyo, Japan) and then transferred to 6- or 96-well plates 1 day before transfection, and Fugene HD (Fugene HD [µL]: DNA [µg]: DMEM [µL] = 5:2:200; Promega, Madison, WI, USA) was used for transient transfection. The transfection mix was incubated for 15 min at room temperature prior to addition to the cell culture in a drop-wise manner (10 µL/well). After indicated time after the transfection, the transfected cells were subjected to further analyses, as indicated below.

### DSP assay and CELISA

To quantify cell–cell fusion, we used the DSP assay, as described previously [28–30, 34, 36]. Briefly, the stable cell line 293FT expressing DSP_1–7_ (293FT/DSP_1–7_), was plated in a 12-well plate (1.5 × 10^5^ cells/well) (BD Falcon, San Jose, CA, USA) with DMEM (Sigma, St. Louis, MO, USA) supplemented with 10% FBS (Hyclone Labs, Logan, UT, USA). 293FT/DSP_1–7_ cells were then transfected with expression vectors of interest in triplicate to generate effector cells. To prepare the target cells, 293CD4/DSP_8–11_ cells, a stable cell line expressing CD4 and DSP_8–11_, were seeded in 6-cm dishes (7.5 × 10^5^/dish) (BD Falcon, San Jose, CA, USA). The DSP assay was performed in triplicate 20 h post-transfection by mixing the effector and target cells at 37 °C in fresh medium containing 60 µM membrane-permeable substrate for RL (Enduren Live Cell Substrate; Promega) added 1 h prior to mixing. *Renilla* activity was measured at 2 h after coculture using a GloMax-Multi Plus Detection System (Promega). Half of the same effector cells were subjected to CELISA to measure the surface expression level of Env. CELISA was performed in a 96-well ViewPlate (PerkinElmer Life Sciences). Briefly, cells were fixed in PBS with 4% paraformaldehyde at 25 °C for 5 min and washed with PBS, then treated with 3% hydrogen peroxide in PBS at 25 °C for 10 min to deactivate endogenous peroxidases. Fixed cells were incubated with 3% ECL Prime Blocking Reagent (GE Healthcare, NY, USA) in PBS at 25 °C for 30 min. After blocking, cells were incubated with a saturating amount of 2G12 (13.1 µg/mL, 1:1000 dilution) at 25 °C for 1 h and then incubated with anti-human IgG-HRP at 1:3000 dilution (Santa Cruz Biotechnology) at 25 °C for 1 h. Between each procedure, cells were washed with PBS three times. Antibodies were diluted in 1.5% ECL Prime Blocking Reagent. Luminescence was detected using ECL Prime Western Blotting Detection Reagent (GE Healthcare) and a GloMax 96 microplate luminometer (Promega). Before calculation of the relative expression levels, the average background signal from blank control wells was subtracted.

### Syncytia formation assay and virus infection assay

Fusion activity was further evaluated by syncytia formation assays. Expression vectors for Env were transfected into 293CD4 cells using FuGENE HD. Unless otherwise indicated, visible syncytia formation was evaluated at 16–24 h post-transfection. To visualize the nuclei of the cells in syncytia formation assays, Hoechst 33,342 (0.2 mg/mL; Invitrogen) was applied at 37 °C for 15 min. After labeling, the cells were washed three times with 200 µL of prewarmed DMEM/FBS, and images were captured using a confocal microscope (Olympus FluoView FV1000). The number of nuclei in syncytia was counted and divided by the total number of the nuclei in the field to estimate the degree of syncytia formation. Five randomly chosen fields were examined.

Pseudotyped virus was generated by transfecting the following plasmids in the ratio of 2:1:1; pLenti-CMV-GFPnluc (reporter expressing GFP-nluc fusion protein), psPAX2 (gag-pol expression), and envelope expression vectors for several gp41 mutants and the wild-type. For the infection assay, the plasmids were transfected into 293FT cells by the calcium phosphate precipitation method. One day after transfection, the medium was replaced and virus-like particles were harvested 2 days after transfection. The virus amount was measured by the p24 ELISA kit (Zeptrometrix, Buffalo, NY), and the same amount of filtered (0.45-µm filter) virus (4.5 ng of p24) was applied to NP2-CD4-CXCR4-DSP1 cells [32] in a 96 well plate (1 × 10^4^ cells/well) in the presence of polybrene (10 µg/mL). The medium was replaced 24 h after infection, and the infectivity of the virus was evaluated at 48 h after infection by measuring n-luc reporter activity. The measurements were taken after incubating infected cells for 1 h in the medium containing 6 µM EnduRen substrate (Promega, WI) using the luminometer (CentroXS3, Berthold Technologies, Bad Wildbad, Germany).

### Synthesis of peptides and CD analysis

The following peptides were synthesized: N36, SGIVQQQNNLLRAIEAQQHLLQLTVWGIKQLQARIL; C34, WMEWDREINNYTSLIHSLIEESQNQQEKNEQELL; and C34 (647+A), WMEWDREINNYTSLIHSLIAEESQNQQEKNEQELL. The peptides were synthetized by GL Biochem (Shanghai) Limited (Shanghai, China) with a purity of more than 95%. CD spectra were acquired on a Chirascan Plus CD spectrometer (Applied Photophysics Limited, Surry, UK) with a thermoelectric sample temperature controller. Samples for wavelength spectra were prepared as 10 µM peptide in phosphate-buffered saline (PBS). The cuvette had a 0.1 cm path length. The wavelength dependence of molar ellipticity, [θ], was monitored at 17 °C as the average of three scans, using a 2.5-s integration time at 1.0-nm wavelength increments. Spectra were baseline-corrected against the value for the cuvette with buffer alone. Thermal stability was determined in the same buffer by measuring [θ]_222_ as a function of temperature. A cell with a path length of 0.1 cm was used with continuous stirring. Thermal melts were monitored in 2 °C intervals with a 2-min equilibration at the desired temperature and an integration time of 30 s. Protein concentrations were determined by measuring the absorbance at 280 nm in the same buffer.

### Immunoblotting

293FT cells (2 × 10^5^) were transiently transfected with Env expression vector using FuGENE HD in a 6-well culture plate. Cells were lysed with 60 µL of RIPA lysis buffer (Thermofisher Scientific, MA, USA) at 48 h after transfection. After centrifugation at 20,000×*g* for 30 min at 4 °C, the supernatant was collected, and the protein concentration was determined by Pierce BCA protein assay (Pierce Biotechnology, Rockford, IL, USA). Samples containing approximately 50 µg of protein were loaded into each well, separated by sodium dodecyl sulfate polyacrylamide gel electrophoresis on 10% gels (Bio-Rad Ready Gel J), and transferred to polyvinylidene fluoride membranes (Immobilon-PSQ; Millipore, MA, USA). The blots were probed with goat anti-gp120 polyclonal antibodies (Fitzgerald, Concord, MA, USA) or the monoclonal antibody Chessie 8 [38]. Donkey anti-goat IgG-horseradish peroxidase (HRP; Santa Cruz Biotechnology, Santa Cruz, CA, USA) or goat anti-mouse IgG-HRP (Santa Cruz Biotechnology) was used as the secondary antibody. The blot was further treated with an ECL Western Blot Kit (CWBIO, Beijing, China). Images were obtained with an LAS3000 system (Fujifilm, Tokyo, Japan). Band intensities were analyzed using ImageJ software (NIH, MD, USA).

For the protein profile analysis of VLPs, the pseudotyped virus was generated by Fugene HD- mediated transfection of 293FT cells with the following plasmids: pLenti-CMV-GFPnluc, psPAX2, and envelope expression vectors (in the ratio of 2:1.5:1). The protein profile of the released VLPs was analyzed by immunoblotting. The VLPs were collected by pelleting down from the cleared and filtered (0.45-µm) culture supernatant of the transfected 293FT cells by ultracentrifugation (20,000 rpm, SW32 rotor, Beckman) at 4 °C for 2 h. The VLPs were lysed in RIPA lysis buffer (Thermofisher Scientific, MA, USA) and subjected to SDS PAGE. The immunoblotting procedure was similar to that for cell lysates as described above. For VLPs, an anti-p24 monoclonal antibody (Santa Cruz Biotechnology, Dallas, TX), was used in addition to anti-Env (gp120 and gp41).

### Statistical analysis

Statistical analyses were performed using Prism software (GraphPad, USA). *T* test was used to compare groups of normalized DSP activity of CHR mutants, syncytia formation assay, and pseudotype virus infection assay. One-way ANOVA test were used to assess the expression level of HIV-1 envelop proteins. Statistical significance was set as *p* < 0.05.

## Additional file

**Additional file 1: Fig. S1.** Interaction of the C34 (647+A) peptide with N36. A. CD spectrographic analysis of the complexes formed between N36 and C34 or its mutant C34 (647+A). Left panel, the secondary structure of complexes formed by C34 and N36 (dark blue) or C34 (647+A) and N36 (orange). Right panel, the stability of complexes formed by C34 with N36 (dark gray) and its mutants (light gray), as measured by thermal denaturation analysis.

## Abbreviations

Env: envelope glycoprotein
HIV-1: human immunodeficiency virus type 1
CHR: C-terminal heptad repeat
NHR: N-terminal heptad repeat
6HB: six-helix bundle
GFP: green fluorescent protein
RL: *Renilla* luciferase
RLU: relative light units
DSP: dual split protein
CD: circular dichroism
MSD: membrane-spanning domain
CT: cytoplasmic
TM: transmembrane
FP: fusion peptide
FBS: fetal bovine serum
PBS: phosphate-buffered saline
