# Supplementary figures and images for: Six-helix bundle completion in the distal C-terminal heptad repeat region of gp41 is required for efficient human immunodeficiency virus type 1 infection
Source: Retrovirology. 2018 Apr 2;15:27. doi: 10.1186/s12977-018-0410-9 (PMC5879932; doi:10.1186/s12977-018-0410-9)

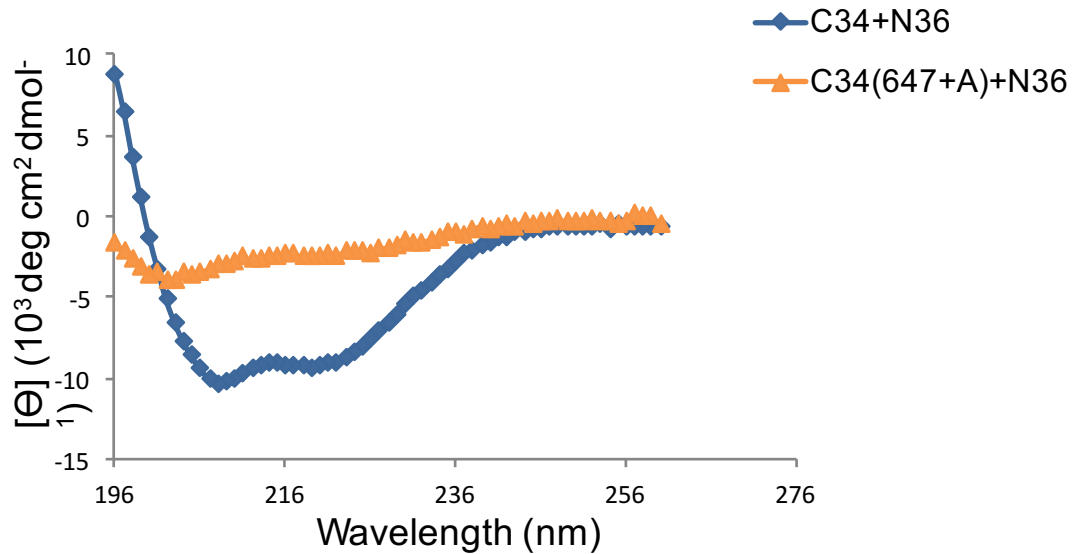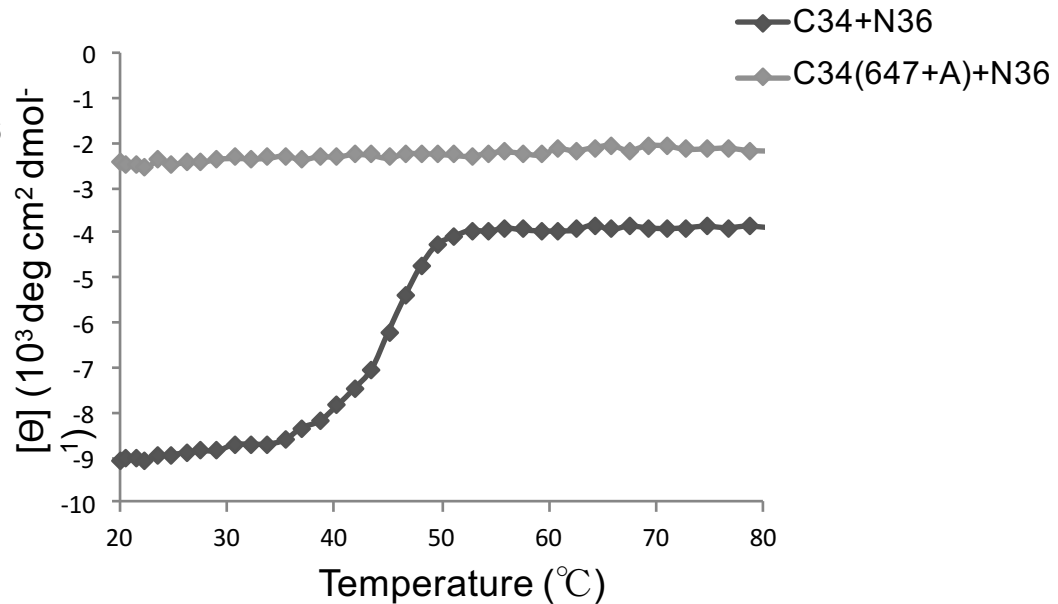

Supplement: Supplementary file 1 — Additional file 1: Fig. S1. Interaction of the C34 (647+A) peptide with N36. A. CD spectrographic analysis of the complexes formed between N36 and C34 or its mutant C34 (647+A). Left panel, the secondary structure of complexes formed by C34 and N36 (dark blue) or C34 (647+A) and N36 (orange). Right panel, the stability of complexes formed by C34 with N36 (dark gray) and its mutants (light gray), as measured by thermal denaturation analysis. [file 12977_2018_410_MOESM1_ESM.pdf]
